# Surgery

**Published:** 1902-06

**Authors:** C. A. Morton


### SURGERY.

In an important paper by Christian Fenger[3] on **Tuberculosis of the Peritoneum**, the author points out the fallacy of concluding that because patients recover in large numbers after laparotomy for this disease, that therefore the operation is the cause of their recovery. He refers to the work of Borchgrevink in connection with the subject,[4] and quotes the conclusion of the writer as follows: "Serous tuberculous peritonitis is a territory which surgery must hand back to the internal medicine clinic, with thanks for the splendid opportunity which a misunderstanding gave to the profession, by means of laparotomy, to study tuberculosis in one of the large cavities of the body."

Borchgrevink has observed twenty-two cases treated by laparotomy and seventeen treated without. He finds the presence of pyrexia adds greatly to the gravity of the case, for out of the twenty-two cases with pyrexia operated on no less than eight died; whereas of those without pyrexia operated on (ten) only one died. Out of the seventeen cases treated without laparotomy fourteen recovered, and of the three cases that died only one died simply from the advance of the tuberculous peritonitis; for one died of intestinal obstruction and another from measles. Borchgrevink's statistics also show that although the fluid returns more frequently after simple puncture than after laparotomy, yet after a time the recurrent collection of fluid is

[3] *Ann. Surg.*, 1901, xxxiv. 771.

[4] *Bibliotheca Medica;* Abtheilung, E.; Chirurgie, Kocher, König and Mikulicz. Heft iv., 1901; quoted by Fenger.

absorbed in most of the cases treated by simple puncture. I believe myself that if it is necessary to evacuate the ascitic fluid in this disease, a small incision is safer than puncture, because the intestine is so liable to be adherent to the anterior abdominal wall, and its presence there may not be made known by resonance. I, however, entirely agree with the author that tuberculous peritonitis is frequently recovered from, and that a great number of cases published as cured by laparotomy would have recovered without. I have frequently seen cases of tuberculous peritonitis with a view to operation, and advised against it, and the patient has recovered. With regard to the method by which laparotomy cures, or is supposed to cure, tuberculous peritonitis, the author refers to the conclusion of Herzfeld,[1] who says, "there is nothing left but to confess that we still stand before an enigma which for the time being cannot be cleared up." Probably there is no enigma to be solved, because the laparotomy has not cured the tuberculous peritonitis; the patient would have recovered without it.

The much discussed question as to whether, when an **appendicitis abscess** is opened, the diseased appendix should be removed has recently been brought prominently before surgeons, by a paper by Mr. Battle[2] advocating its removal either when the abscess is opened, or after convalescence; and one by Mr. Gilbert Barling[3] showing how rarely trouble follows if the appendix is not removed. Mr. Battle records seven cases to show what trouble may follow if the appendix is left. In three of these one or more abscesses formed after the first had healed, in two there were recurrent attacks with suppuration, in one a sinus, and in another a fæcal fistula persisted. But he does not tell us in how many of his cases in which the appendix was not removed, no trouble subsequently arose. Mr. Barling, on the other hand, records forty-seven cases (all traced) of appendix abscess in which the appendix was not removed, and of these cases only one had recurrent trouble. The report of Mr. Barling's paper, however, does not inform us whether any of his cases had persistent sinus after operation, though they did not get recurrent attacks. In the discussion that ensued on Mr. Barling's paper,[4] Mr. Wallace stated that hospital (? St. Thomas') statistics for six years showed about 5 per cent. of recurrent suppuration after drainage of the appendix abscess without removal of the appendix. Mr. Barker expressed an opinion that the appendix should either be removed at the time the abscess is opened or after convalescence. I have had a sinus persist for some time after the abscess has been opened in some of my cases. In one case I have

[1] *Mitth. a. d. Grenzgebeiten d. Med. u. Chir.*, 1900, Bd. v., Heft ii., 1884; quoted by Fenger.

[2] *Lancet*, 1902, i. 143.

[3] *Brit. M. J.*, 1902, i. 455.

[4] *Loc. cit.*, 456.

had to remove the appendix many weeks after the abscess was drained, in order to stop the persistent suppuration, and in another such persistent suppuration proved fatal as the patient refused further operation. I believe I am correct in saying that I have only had one case of recurrent abscess[1] (I have not time at present to look through my records), but in another case the patient had one or two recurrent non-suppurative attacks after the drainage of the abscess; and in one case peritonitis followed the healing of a sinus after the drainage of an appendicitis abscess, from recurrent mischief in the appendix. My own opinion is that it is a very risky thing to break down the adhesions amongst the coils of intestines which form the walls of the abscess, in order to remove the appendix, for in this way we may infect the general peritoneal cavity; and the proportion of cases in which trouble remains (persistent suppuration) or follows the drainage of the abscess (recurrent attacks) is too small to warrant us in incurring the risk, or in performing a second operation to remove the appendix after recovery.

In this *Journal* in 1897 I referred to the treatment of paralytic club-foot by **tendon grafting.** In 1898 Mr. Eve published a report of cases treated by this method,[2] and more recently Mr. Tubby and Mr. Sinclair White have done the same.[3] Dr. Fritz Lange has also published a recent paper on the subject.[4] The method consists in transplanting the tendon of an active muscle on to the tendon of a paralysed muscle. For instance, the tibial muscle may be paralysed, and the other muscles of the foot and leg may escape. The active muscles will produce deformity by overaction, as they will not be opposed by the paralytic muscle. Hence a paralytic valgus may be produced. The tendon of the extensor proprius hallucis may be divided, and also the tendon of the paralysed tibialis, and the proximal end of the extensor tendon securely sutured to the distal portion of the tibialis anticus tendon. Various tendons have been transplanted in this way. Sometimes a strip has been taken from the side of the tendo-achillis and attached to some paralysed muscle. The result has generally been good. If the tendon or tendons transplanted are functionally allied to the tendon of the paralysed muscle to which they are attached, the return of voluntary power to perform the movements of the paralysed muscle may be anticipated. If the transplanted muscle is one of an antagonistic group, a reflex contraction on walking may be expected, and this will tend to hold the foot in an improved position. Moreover, the loss of power in the overacting group of muscles due to the transplantation of one or more of them will diminish the

[1] The case of persistent fatal suppuration.

[2] *Brit. M. J.*, 1898, ii. 1139. [3] *Ibid.*, 1901, ii. 585, *et seq.*

[4] *München. med. Wchnschr.*, 1902, xlix. 10.

tendency to deformity. If the tendons on one side of the foot have to be attached to those on the other, it is not necessary to carry a long incision over the foot. A short incision can be made over the tendon or tendons on each side, and they can be carried across the foot by tunnelling under the tissues by means of a pair of sinus forceps or a pair of blunt-pointed scissors.

Lange[1] has shown that in cases in which the transplanted tendon is not long enough to reach to the place to which we wish to attach it, it may be joined by a silk suture, carried on from the tendon and acting as a prolongation of it. If the tendon of the paralysed muscle is much atrophied, the author thinks it may stretch and become useless, and prefers to attach the tendon of the active muscle by a silk cord to the periosteum at the insertion of the paralysed tendon. He draws attention to the risk to the vitality of the tendon of tension in the union, and advises the use of a silk suture as a prolongation of the tendon in cases in which the tendon itself can only be attached in a condition of tension. Lange has employed this method in fifty-six cases, with good results in nearly all. The silk tendons were rendered aseptic with great care, and never produced any suppuration. It is a most interesting fact that in one case the author found that after a time new fibrous tissue formed around the silk cord, which appeared as bluish-white, tough, fibrous cord, of the size of a large lead-pencil. There was around it a layer of loose and movable connective tissue, but a true tendon sheath had not formed.

C. A. MORTON.

